# Effects of an Early Postnatal Music Intervention on Cognitive and Emotional Development in Preterm Children at 12 and 24 Months: Preliminary Findings

**DOI:** 10.3389/fpsyg.2019.00494

**Published:** 2019-03-05

**Authors:** Fleur Lejeune, Lara Lordier, Marie P. Pittet, Lucie Schoenhals, Didier Grandjean, Petra S. Hüppi, Manuela Filippa, Cristina Borradori Tolsa

**Affiliations:** ^1^Child Clinical Neuropsychology Unit, Faculty of Psychology and Education Sciences, University of Geneva, Geneva, Switzerland; ^2^Division of Development and Growth, Department of Pediatrics, Geneva University Hospitals, Geneva, Switzerland; ^3^Swiss Center for Affective Sciences, University of Geneva, Geneva, Switzerland; ^4^Neuroscience of Emotion and Affective Dynamics Lab, Faculty of Psychology and Educational Sciences, University of Geneva, Geneva, Switzerland

**Keywords:** preterm children, early intervention, music, anger reactivity, fear reactivity, emotion regulation

## Abstract

Preterm birth is associated with a higher prevalence of neurodevelopmental deficits. Indeed, preterm children are at increased risk for cognitive, behavioral, and socio-emotional difficulties. There is currently an increasing interest in introducing music intervention in neonatal intensive care unit (NICU) care. Several studies have shown short-term beneficial effects. A recent study has shown that listening to a familiar music (heard daily during the NICU stay) enhanced preterm infants’ functional connectivity between auditory cortices and subcortical brain regions at term-equivalent age. However, the long-term effects of music listening in the NICUs have never been explored. The aim of this study was to evaluate at 12 and 24 months the effects of music listening in the NICU on cognitive and emotional development in preterm children by comparing them to a preterm control group with no previous music exposure and to a full-term group. Participants were 44 children (17 full-term and 27 preterm). Preterm children were randomized to either music intervention or control condition (without music). The preterm-music group regularly listened to music from 33 weeks postconceptional age until hospital discharge or term-equivalent age. At 12 months, children were evaluated on the Bayley Scales of Infant and Toddler Development, Third Edition, then with 4 episodes of the Laboratory Temperament Assessment Battery (assessing expressions of joy, anger, and fear, and sustained attention). At 24 months, the children were evaluated with the same tests, and with 3 additional episodes of the Effortful Control Battery (assessing inhibition). Results showed that the scores of preterm children, music and control, differed from those of full-term children for fear reactivity at 12 months of age and for anger reactivity at 24 months of age. Interestingly, these significant differences were less important between the preterm-music and the full-term groups than between the preterm-control and the full-term groups. The present study provides preliminary, but promising, scientific findings on the beneficial long-term effects of music listening in the NICU on neurodevelopmental outcomes in preterm children, and more specifically on emotion mechanisms at 12 and 24 months of age. Our findings bring new insights for supporting early music intervention in the NICU.

## Introduction

Numerous studies reported a higher prevalence of neurodevelopmental deficits in children born prematurely compared to full-term children. More specifically, preterm survivors are at increased risk for cognitive (Bhutta, 2002; Brydges et al., 2018), behavioral (Bröring et al., 2018; Franz et al., 2018), and socio-emotional difficulties (Clark et al., 2008; Treyvaud et al., 2012; Lejeune et al., 2016; Montagna and Nosarti, 2016) which can negatively impact on their academic achievements (Akshoomoff et al., 2017; Twilhaar et al., 2018) and tend to persist into adolescence and adulthood (Hack et al., 2002; Cooke, 2004; Indredavik et al., 2005; Linsell et al., 2018). From an early age, emotional, attentional and inhibition impairments are frequently reported in preterm infants (Anderson et al., 2011; Aarnoudse-Moens et al., 2012). Interestingly, some researchers followed the developmental trajectory of these abilities longitudinally in a cohort of very preterm infants in comparison to their full-term peers. At 12 months, preterm infants exhibited greater reactivity to anger and lower reactivity to fear than full-term infants (Langerock et al., 2013). At 24 months, they were described by their parents as having a higher level of negative affect (Lejeune et al., 2015). At 42 months, they had higher scores of frustration and fear levels, and were less accurate when naming emotional facial expressions, including happiness, sadness, fear, anger and disgust (Witt et al., 2014). In addition, 12- and 24-month-children infants showed distinct attentional patterns compared to full-term children (Langerock et al., 2013; Lejeune et al., 2015). Preterm children also exhibited early inhibition difficulties compared to their full-term peers at 24 and 42 months (Witt et al., 2014; Lejeune et al., 2015). These studies highlight the necessity of implementing early intervention to support cognitive and emotional development in preterm infants.

In the absence of major brain lesions, these neurodevelopmental difficulties are both due to the disruption of normal brain development and to prematurity itself, as well as to an adverse postnatal environment. Preterm birth interrupts abruptly the brain maturation and can result in delayed or abnormal brain development during critical periods involving glial cell proliferation, synaptogenesis, pruning, and initiation of myelination (Volpe, 2001). Therefore preterm infants are at high risk for injury to the gray and white matter (Inder et al., 1999), delay in cortical maturation (Dubois et al., 2008), brain tissue volume alterations (Peterson et al., 2003; Nosarti et al., 2004; Inder et al., 2005; Mewes et al., 2006; Keunen et al., 2012; Cismaru et al., 2016), as well as impaired connectivity with long-term effects on socio-emotional and cognitive outcomes (Peterson et al., 2000; Treyvaud et al., 2013; Matthews et al., 2018). Moreover, preterm infants are exposed for weeks in the neonatal intensive care unit (NICU), to important factors of stress such as an atypical sensory environment (including high levels of noise and light), maternal separation and exposure to routine pain procedures. All these factors have short-term effects with behavioral and physiological stress responses (Peng et al., 2009), as well as long-term effects on their emotional and cognitive development (Montagna and Nosarti, 2016). For example, numerous studies have focused on the negative effect of noise and found that intense sounds act as stressful events on physiological self-regulatory abilities (Wachman and Lahav, 2011). The stress generated by these inadequate sensory stimulations leads to significant changes in the hypothalamic-pituitary-adrenal axis, as well as changes in brain development which could in turn impact the subsequent neurodevelopmental outcomes of preterm infants (Mooney-Leber and Brummelte, 2017).

In this context, there is currently a major need of developing intervention which aims to support the sensory and emotional development of preterm newborns by offering them a physical and human environment adapted to their needs. Developmental care programs are designed to limit overstimulations, pain and stress for preterm infants in the NICU, and to promote their well-being through various interventions, such as reduction of light and sound, skin-to-skin contact or massage therapy. These programs have already shown positive effects on neurodevelopmental outcomes in children born preterm (Spittle and Treyvaud, 2016). Environmental enrichment by music might be a non-invasive intervention to reduce preterm infants’ stress during their hospitalization in the NICU.

Listening to music is a complex cerebral process, as it involves auditory, cognitive, motor, and emotional functions soliciting widespread activation of various neuronal networks (Koelsch, 2014; Sihvonen et al., 2017). Studies showed that listening to music had positive effects on stress and anxiety reduction in healthy adults (Linnemann et al., 2015a; Panteleeva et al., 2018) and newborns (Rossi et al., 2018), as well as for pain-reduction in patients with chronic pain disease (Linnemann et al., 2015b) or in postoperative patients who had various types of major surgery (Hole et al., 2015). These studies suggest that music intervention may enhance self-regulatory abilities. Music seems to be a relevant intervention in the management of stress, anxiety and pain in vulnerable population, such as preterm newborns, which could in turn have positive effects on their long-term neurodevelopment (see for a review, Anderson and Patel, 2018).

Recent studies have considered the effects of music listening in preterm infants and many have shown that proposing harmonious and regular sounds had short-term beneficial effects (during NICU stay and until hospital discharge), such as stabilizing heart and respiratory rate, reducing apnea or bradycardia episodes, improving resting energy expenditure and feeding, better weight gain and more mature sleep patterns (Anderson and Patel, 2018). Music listening has been shown to activate brain regions related to emotional processing in adults (Koelsch, 2014) and even in full-term newborns (Perani et al., 2010). Furthermore, a recent study has shown that repeated listening to familiar music (heard daily during the NICU stay) enhanced the functional connectivity of preterm infants between the auditory cortices and the subcortical brain regions at term-equivalent age. This result might not only reflect that preterm infants recognized the known music but also that they perceived it as more arousing and pleasant (Lordier et al., 2018). Other studies have reported positive effects on behavioral development after exposure to a breathing bear (Thoman et al., 1991; Ingersoll and Thoman, 1994), to the sound of a heartbeat (Barnard and Bee, 1983), or to voices (Nöcker-Ribaupierre et al., 2015; Filippa et al., 2017; Best et al., 2018; Saliba et al., 2018) during the NICU stay. However, the long-term effects of music listening in the NICUs on preterm infant’s cognitive and emotional development have never been explored so far.

Self-regulatory abilities, which are impaired in the preterm survivors (Langerock et al., 2013; Witt et al., 2014; Lejeune et al., 2015), have be shown to be enhanced by music listening in different clinical population, such as patients with chronic pain disease or those who had various types of major surgery (Hole et al., 2015; Linnemann et al., 2015b), as well as in full-term newborns (Rossi et al., 2018). Assessment of the long-term effect of music interventions in preterm children should focus on these specific outcomes.

The aim of this study was to evaluate long-term effects of music listening in the NICU on cognitive and emotional development in preterm children by comparing them to a preterm control group with no previous music exposure and to a full-term group at 12 and 24 months. The cognitive and emotional abilities of the preterm music group were expected to be higher than those of the preterm control group, as well as to be closer to those of the full-term group.

## Materials and Methods

### Participants

The initial cohort included 39 preterm infants (gestational age at birth < 32 weeks) and 24 full-term infants, born between March 2013 and October 2015, who were participants in a longitudinal study assessing the effects of early music exposure during the NICU stay on brain processing (Lordier et al., 2018) and neurobehavioral development. Infants were recruited at the neonatal units of the University Hospital of Geneva. Written informed parental consent was obtained for each newborn prior to participation. The study was conducted in accordance with the Declaration of Helsinki and approved by the ethics committee of the University Hospital of Geneva.

**FIGURE 1:**
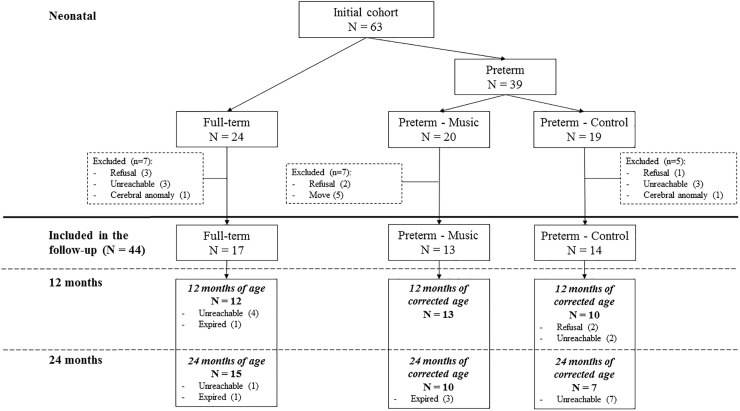
Flow chart of participants in the study.

The present study concerned the cognitive and emotional evaluation of these children at 12 and 24 months of age, which took place in the follow-up unit of the University Hospital of Geneva. Nineteen participants (7 full-term and 12 preterm) from the initial cohort did not participate in the follow-up assessment (Figure 1). Exclusion criteria for all newborns were major brain lesions detected on early MRI, such as intraventricular hemorrhage grade III with or without apparent periventricular hemorrhagic infarction, or cystic periventricular leukomalacia. The final sample consisted of 44 children (17 full-term and 27 preterm). There was no significant difference in demographic and perinatal variables between full-term children who participated in the follow-up and those who dropped out. For the preterm group, there was only one significant difference for the family’s socioeconomic status (SES), *t*(35) = 2.760, *p* = 0.009. The family’s SES is a 12-point scale based on paternal occupation and maternal education (range from 2 – the highest SES – to 12 – the lowest SES). The SES of the families of preterm children who dropped out (mean = 3.20, *SD* = 0.9) was higher than that of the families of preterm children who participated in the follow-up assessment (mean = 6.22, *SD* = 3.4).

Preterm infants were randomized to either music intervention or control condition (without music). Figure 1 illustrates the flow chart of the participants in the study. The preterm-music group consisted of 13 children at 12 months (mean corrected age = 14.7 months, SD = 2), and 10 children at 24 months (mean corrected age = 24.9 months, *SD* = 1.03). The preterm-control group consisted of 10 children at 12 months (mean corrected age = 14 months, *SD* = 1.2), and 7 children at 24 months (mean corrected age = 25.6 months, *SD* = 1.2). The full-term group was composed of 12 children at 12 months (mean age = 14.8 months, *SD* = 1.9), and 15 children at 24 months (mean age = 25.8 months, *SD* = 1.9). The mean ages of the three groups did not differ significantly at both assessment ages (all *ps* > 0.05). The SES of the families of preterm children (mean = 6.22, *SD* = 3.4) was marginally lower than that of the families of full-term children (mean = 4.47, *SD* = 2.8), *t*(42) = 1.857, *p* = 0.07.

Table 1 presented demographic and perinatal variables of the three groups. The preterm-music and the preterm-control groups did not differ significantly on these demographic and perinatal variables.

**Table 1:** Population characteristics.

	Full-term	Preterm-control	Preterm-music	Preterm-control vs. Preterm-music
	*n* = 17	*n* = 14	*n* = 13	
				
	*n*(%) or mean (*SD*)	*n*(%) or mean (*SD*)	*n*(%) or mean (*SD*)	*p*-value^a^
Sex, number of girls	9 (52.9)	7 (50)	8 (61.5)	0.547
Socioeconomic score^b^	4.47 (2.8)	6.07 (3.3)	6.38 (3.6)	0.867
Gestational age at birth (weeks)	39.57 (1)	29 (2.2)	29.14 (2.3)	0.905
Birth weight (g)	3271 (410)	1207 (274)	1217 (377)	1.000
Small for gestational age^c^	1 (5.9)	3 (21.4)	1 (7.7)	0.315
Birth height (cm)	49.3 (1.4)	37.2 (4)	38 (3.4)	0.519
Birth head circumference (cm)	34.2 (1.3)	26.6 (2.2)	27.1 (3.1)	0.519
Broncho-pulmonary dysplasia	0	5 (35.7)	2 (15.4)	0.228
Intraventricular hemorrhage (grade I–II)	0	3 (21.4)	4 (30.8)	0.580
Early and late onset sepsis	0	3 (21.4)	0	0.077
Patent ductus arteriosus	0	1 (7.1)	1 (7.7)	0.957

*^a^Pearson’s chi-square and Mann–Whitney U-tests were used for the comparison of the variables between the preterm-music and preterm-control groups. ^b^The socioeconomic status (SES) was calculated using the Largo et al. (1989) 12-point scale based on paternal occupation and maternal education (range from 2 – the highest SES – to 12 – the lowest SES). ^c^Small for gestational age:<10th percentile for birth weight as a function of gestational age and gender.*

### Procedure

#### Music Intervention

Preterm infants were randomized to either music intervention (preterm-music) or control condition (preterm-control group). Parents, caregivers and music intervention providers were blind to group assignment. The preterm-music group listened to a music especially created by Andreas Vollenweider^1^ during 8 min with headphones, from gestational age of 33 weeks until hospital discharge or term-equivalent age. The music was composed of background, bells, harp, and punji. Three tracks were created in collaboration with a nurse specialized in developmental care. Music was presented to the baby according to the state of wakefulness, following his biological rhythm: one was composed with the aim of helping the baby to wake up, one to maintain the child in a state of calm awakening, and the last one to help the baby to fall asleep. The nurse determined the readiness for music exposure and chose the track based on a neonatal behavioral assessment scale (Martinet et al., 2013). The intervention was performed only when the baby was lying in the bed. The music extract presented a high degree of homogeneity and repetitions, and it was structured by a continuous background, with short and repetitive melodies in a reduced pitch range. The sound level ranged from 30 dBA (background) to 65 dBA (peak with the bells).

Preterm-music infants listened to music about 5 times per week and preterm-control infants had open headphones put on without music at the same frequency. The mean number of music listening was 24.58 times (*SD* = 9.49) for the preterm-music group and the mean number of having open headphones was 23 times (*SD* = 6.28) for the preterm-control group. More details about the music intervention can be found in Lordier et al. (2018).

Full-term children were recruited at the maternity of the same hospital where they underwent magnetic resonance imaging including an fMRI music paradigm in their first days of life (Lordier et al., 2018). They were thus exposed to music only once during this fMRI. They were then contacted for follow-up assessments at 1 and 2 years.

#### Cognitive and Emotional Evaluation

The children were tested individually in a quiet room with at least one reference person present during a 1-h session. They were seated on the reference person’s lap, or in front of a small table on a small chair. All the evaluations were videotaped with written informed parental consent for subsequent analysis and were done by trained psychologists or developmental pediatricians who were blind to the music group assignment. At 12 months, children were evaluated on the Bayley Scales of Infant and Toddler Development, Third Edition (BSID-III; Bayley, 2006), then with 4 episodes of the Laboratory Temperament Assessment Battery (Lab-TAB; Goldsmith and Rothbart, 1999). At 24 months, the children were evaluated with the same tests, as well as with 3 additional episodes of the Effortful Control Battery (Kochanska et al., 2000). The 4 episodes from the Lab-TAB were Puppet game, Attractive toy placed behind barrier, Unpredictable mechanical toy, and Blocks, assessing expression of joy, anger, fear, and sustained attention, respectively. The 3 episodes from the Effortful Control Battery were Snack delay, Wrapped gift, and Tower, measuring the child’s ability to delay (wait for a pleasant event) twice and to suppress or initiate activity to signal (take turns), respectively. Two coders scored the episodes independently for 16% of the sample after thorough training on the scoring methods. Inter-rater reliability was calculated using Pearson correlations on the means for each variable by episode. Correlations ranged from 0.59 to 1 with a mean *r* of 0.83.

### Measures

#### Bayley Scales of Infant and Toddler Development, Third Edition (BSID-III)

The BSID-III is a standardized battery of tests that assesses development of different domains in 1- to 42-month-old children and generates scores for 3 composite indices (cognitive, language, and motor). Raw scores were converted into standard scores based on adjusted age.

#### Laboratory Temperament Assessment Battery (Lab-TAB)

Lab-TAB coding involves facial, vocal, and bodily measures. For each episode, the measures were coded on a scale from 0 to 3. A higher score indicates higher emotional reactivity.

The puppet game was used to assess joy and involved the presentation of a scripted puppet show lasting about 1 min. This episode consisted of presenting 2 puppets who interacted with the child and tickled him three times. Scoring was performed in four equivalent time intervals (introduction, first tickle, second tickle, and third tickle). For each time interval, intensity of smiling, positive vocalizations and positive motor activity were coded. These scores were averaged to compute a score of Joy.

The attractive toy placed behind barrier was used to assess anger. It consisted of presenting an attractive toy to the child. Once he was playing with interest, the toy was gently removed from his hand and placed behind a transparent Plexiglas barrier for 30 s (2 trials). Scoring for each trial was performed in 6 time intervals of 5 s. For each time interval, intensity of facial anger, distress vocalizations and struggle were coded. These scores were averaged to compute a score of Anger for each trial.

The unpredictable mechanical toy was chosen to elicit fear and involved the presentation of a mechanical robot placed on a table in front of the child. The robot went toward the child, stopped in front of him and barked, and then moved back. The episode lasted 15 s and included 2 trials. Scoring for each trial was performed in 3 time intervals of 5 s. For each time interval, intensity of facial fear, distress vocalizations, bodily fear, escape, and startle response were coded. All measures were then averaged across time intervals to compute a score of Fear for each trial.

The Blocks episode measures sustained attention. The child played freely with decorated cubes for 3 min. Each minute was divided into 6 time intervals of 10 s. Each time interval was coded for intensity of facial interest, duration of observation and duration of manipulation. All scores were averaged to compute a composite score of Sustained attention.

#### Effortful Control Battery

The Snack Delay measures the child’s ability to delay gratification. Children were asked to place their hands on a mat on the table and not to touch or eat a treat placed in front of them under a transparent cup until the experimenter rang a bell (4 trials, with delays of 10, 20, 30, and 15 s, respectively). In the middle of each trial, the experimenter picked up the bell but did not ring it. For each trial, an inhibition score was computed on a scale from 1 to 9 (1 = child eats snack before experimenter lifts bell; 4 = child touches snack after experimenter lifts bell; 7 = child waits until bell rung). One point was added for keeping hands on the mat only before or after the experimenter lifted the bell, and 2 points were added for keeping hands on the mat during the entire trial.

The Wrapped Gift also measures the child’s ability to delay gratification. During the first part of the episode, children were told that they would receive a gift but that they could not peek while the gift was being wrapped. The experimenter asked the child to sit down with his or her back to him as he noisily wrapped (60 s). During the second part, the wrapped gift was placed on the table, and the child was told to stay on his or her chair and not to touch the gift until the experimenter returned with a bow (180 s). Scoring during the first part corresponded to the Turn score on a scale from 1 to 5 (1 = child turns around and continues to peek; 3 = child peeks over shoulder; 5 = child does not peek). Scoring during the second part was divided into 2 scores on a scale from 1 to 4: the touch score (1 = child opens gift; 4 = child never touches the gift) and the Seat score (child is on the seat for a total time of 1 = less than 30 s; 2 = less than 1 min; 3 = less than 2 min; 4 = more than 2 min).

The Tower assesses the ability to take turns by suppressing an impulsive motor response. Children were invited to take turns with the experimenter to build a tower with wooden blocks. The experimenter demonstrated turn-taking to ensure that the child understood what it meant. The episode included 2 trials and an Inhibition scores was computed for each trial. The total number of the placed blocks (multiplied by 10) was divided by the number of blocks put by the child. If a child took turns with the experimenter every time, she or he placed as many blocks as the experimenter (20 was the highest score). A penalty of −5 points was given for intentionally knocking down the tower.

### Statistical Analysis

All statistical analyses were conducted using SPSS 25.0 (IBM SPSS Statistics, IBM Corporation). Kolmogorov–Smirnov analyses were performed to verify the normality of the data. The not normally distributed scores were then transformed into rank-ordered scores (Conover and Iman, 1982). Moreover, all of the analyses were performed to control for the effects of between-group differences in SES, as well as for the age at assessment (chronological age for full-term children; corrected age for preterm children).

Analyses of covariance (ANCOVAs) were run on raw scores for the normally distributed data and on the ranked dependent variables for the not normally distributed ones. These analyses were performed for each dependent variable with the group (preterm music vs. preterm control vs. full-term) as the between-subjects factor. For the tasks comprising several trials, repeated-measures ANCOVAs were conducted with the trial (trial 1 vs. trial n) as the within-subjects factor and the group (preterm music vs. preterm control vs. full-term) as the between-subjects factor. To further investigate the significant group effects, contrasts were conducted. Effect sizes for the overall ANCOVAs were reported (calculated by the SPSS software), as well as those for the contrasts [calculated according to Field (2009), p.390], using the values of *t* and *df*). The significant threshold was 0.05 and the marginal threshold was 0.07.

## Results

### At 12 Months

The results of the evaluation of children at the age of 12 months are presented in Table 2.

**Table 2:** Results of the Bayley and the Lab-TAB at 12 months according to the group.

	Preterm-control *n* = 10	Preterm-music *n* = 13	Full-term *n* = 12		
			
	mean (*SD*)	mean (*SD*)	mean (*SD*)	*F*	*p*
**Bayley scale**					
Cognitive	106 (11)	100.4 (9.7)	110 (9.5)	1.817	0.180
Motor	100.2 (13.2)	99.3 (5.7)	105.7 (9.8)	0.708	0.501
Langage	97.2 (9.5)	95.5 (8.2)	99.7 (5.9)	0.385	0.684
**Puppet game**					
Joy	1.06 (0.5)	0.99 (0.5)	0.88 (0.5)	0.229	0.797
**Toy behind barrier**					
Anger-Trial 1	0.78 (0.7)	0.68 (0.4)	0.72 (0.5)	0.260	0.773
Anger-Trial 2	0.57 (0.4)	0.56 (0.7)	0.84 (0.5)	0.745	0.485
**Unpredictable toy**					
Fear-Trial 1	0.41 (0.4)	0.27 (0.3)	0.42 (0.2)	0.099	0.905
Fear-Trial 2	0.18 (0.2)^∗^	0.35 (0.4)^§^	0.63 (0.3)^§ ∗^	5.612	**0.011**
**Blocks**					
Sustained attention	2.09 (0.6)	2.03 (0.6)	2.17 (0.8)	0.979	0.387

*Boldface entries indicate significant difference (p < 0.05). ^§,∗^Significant contrasts between two groups.*

#### Bayley Scale

No significant group effect was observed.

#### Lab-TAB

For the unpredictable toy episode, results revealed a significant group × trial interaction, *F*(2,21) = 10.12, *p* < 0.001, $\eta_{p}^{2}$ = 0.49. A significant group effect was only observed for the second trial (Anger-Trial 2), *F*(2,21) = 5.612, *p* = 0.011, $\eta_{p}^{2}$ = 0.35. Contrasts indicated that the full-term group had a higher score of fear than the preterm-control group [*F*(1,21) = 10.93, *p* = 0.003, $\eta_{p}^{2}$ = 0.59] and the preterm-music group [*F*(1,21) = 4.63, *p* = 0.043, $\eta_{p}^{2}$ = 0.42] during the second trial, showing that the difference of fear reactivity was less important between the preterm-music and the full-term groups ($\eta_{p}^{2}$ = 0.42) than between the preterm-control and the full-term groups ($\eta_{p}^{2}$ = 0.59). Contrasts also showed that a significant increase in fear reactivity was observed between Trial 1 and Trial 2 in the full-term group [*F*(1,21) = 12.03, *p* = 0.002, $\eta_{p}^{2}$ = 0.60], whereas the opposite was observed in the preterm-control group [*F*(1,21) = 7.05, *p* = 0.015, $\eta_{p}^{2}$ = 0.50]. There was no significant change in fear reactivity between the two trials in the preterm-music group [*F*(1,21) = 0.34, *p* = 0.565, $\eta_{p}^{2}$ = 0.13]. No other significant effect was observed.

### At 24 Months

The results of the evaluation of children at the age of 24 months are presented in Table 3.

**Table 3:** Results of the Bayley, the Lab-TAB and the Effortful Control Battery at 24 months according to the group.

	Preterm-control *n* = 7	Preterm-music *n* = 10	Full-term *n* = 15		
			
	mean (*SD*)	mean (*SD*)	mean (*SD*)	*F*	*p*
**Bayley scale**					
Cognitive	98.6 (6.9)	100 (10.8)	101.7 (7.9)	0.015	0.985
Motor	102.1 (8.1)	101.5 (9.7)	106.3 (13.5)	0.555	0.581
Langage	92.1 (8.5)	92.1 (8.4)	95.9 (12)	0.283	0.756
**Puppet game**					
Joy	0.83 (0.6)	0.87 (0.6)	0.98 (0.6)	0.240	0.789
**Toy behind barrier**					
Anger-Trial 1	0.49 (0.3)	0.49 (0.3)	0.68 (0.4)	1.240	0.308
Anger-Trial 2	0.44 (0.4)^∗^	0.51 (0.3)^§^	0.82 (0.4)^§ ∗^	3.659	**0.042**
**Unpredictable toy**					
Fear-Trial 1	0.74 (0.4)	0.77 (0.6)	0.76 (0.6)	0.093	0.912
Fear-Trial 2	0.76 (0.5)	0.88 (0.8)	0.98 (0.7)	0.861	0.438
**Blocks**					
Sustained attention	2.31 (0.4)	2.49 (0.3)	2.59 (0.3)	1.457	0.254
**Snack delay**					
Inhibition-Trial 1	9 (0)	8.2 (1.5)	7.27 (2.3)	1.881	0.178
Inhibition-Trial 2	7 (4)	7.2 (2.8)	7.45 (2.8)	0.075	0.928
Inhibition-Trial 3	6.75 (3.9)	7.1 (2.5)	7.2 (2.7)	0.110	0.896
Inhibition-Trial 4	8 (2)	6.6 (2.7)	7.82 (1.7)	0.774	0.475
**Wrapped gift**					
Peak and turn	2.83 (1.8)	1.44 (0.7)	1.86 (1.4)	2.071	0.148
Touch	3.33 (1)	3.33 (1.1)	3.29 (1)	0.011^a^	0.989
Seat	2.33 (1.5)	2 (1.3)	2.14 (1.1)	0.099	0.906
**Tower**					
Inhibition-Trial 1	16.6 (2.3)	17.2 (1.6)	14.8 (2)	2.138	0.157
Inhibition-Trial 2	18.1 (2.1)	17.1 (1.7)	15.9 (2.8)	1.719	0.218

*Boldface entries indicate significant difference (p < 0.05). ^a^Normalized by rank-ordered transformation for ANCOVAs. ^§,∗^Significant contrasts between two groups.*

#### Bayley Scale

No significant group effect was observed.

#### Lab-TAB

For the toy behind barrier episode, results revealed a significant group effect for the second trial (Anger-Trial 2), *F*(2,23) = 3.659, *p* = 0.042, $\eta_{p}^{2}$ = 0.24. Contrasts indicated that the full-term group had a higher score of anger than the preterm-control group [*F*(1,23) = 5.988, *p* = 0.022, $\eta_{p}^{2}$ = 0.45] and the preterm-music group [*F*(1,23) = 4.26, *p* = 0.05, $\eta_{p}^{2}$ = 0.39] during the second trial, showing that the difference of anger reactivity was less pronounced between the preterm-music and the full-term groups ($\eta_{p}^{2}$ = 0.39), than between the preterm-control and the full-term groups ($\eta_{p}^{2}$ = 0.45). No other significant effect was observed.

#### Effortful Control Battery

No significant effect between groups was observed.

## Discussion

The present study aimed to assess the effects of music listening in the NICU at 12 and 24 months of age on cognitive and emotional abilities in preterm children by comparing them to a preterm control group with no previous music exposure and to a full-term group. Results showed that the scores of the two groups of preterm children, music and control, differed from those of the full-term children for fear reactivity at 12 months of age and for anger reactivity at 24 months of age. Interestingly, these significant differences were less important between the preterm-music and the full-term groups than between the preterm-control and the full-term groups. These results will be discussed in regards to music listening in the NICU.

Firstly, during the fear-eliciting episode of the Lab-TAB, the full-term group aged 12 months expressed a higher level of fear reactivity than the preterm-control and the preterm-music groups during the second trial. This result is in accordance with previous findings showing that 12-month-old preterm infants perceived the unpredictable mechanical dog episode as less frightening than did the full-terms (Langerock et al., 2013). Furthermore, we did not find any difference at 24 months of age. In line with these findings, Lejeune et al. (2015) showed no difference between 24-month-old preterm and full-term children using the same fear-eliciting episode. However, at 42 months, preterm children displayed higher fear reactivity during another fear-eliciting episode of the Lab-TAB (the mask) than their full-term peers (Witt et al., 2014). This different developmental trajectory of fear reactivity in the two populations is in favor of a developmental delay hypothesis in the preterm population.

Interestingly, the effect sizes also revealed that the difference of fear reactivity was less important between the preterm-music and the full-term groups ($\eta_{p}^{2}$ = 0.42) than between the preterm-control and the full-term groups ($\eta_{p}^{2}$ = 0.59). Moreover, a significant increase in fear reactivity was observed between Trial 1 and Trial 2 in the full-term group, while the opposite was observed in the preterm-control group. The preterm-music group did not show any significant change in fear reactivity between the two trials. Taken together, these results suggest that music exposure in NICU would have a positive impact on fear processing. Recently, a study has revealed a processing bias toward fear: when preterm adults were presented with different facial emotion and they had to identify the emotion, they were more likely to report fear than another negative emotion (Gao et al., 2017). The authors suggested that this bias for fear could reflect a dysregulation of the neuronal distributed fear system. Our results showing that the processing of fear in the preterm-music group is closer to that of the full-term group, is promising as music listening in the NICU could have long-term positive effects on fear processing and regulation.

Cismaru et al. (2016) compared amygdala volumes in full-term infants and preterm infants at term-equivalent age, and related preterm infants’ amygdala volumes with their performance on the unpredictable mechanical dog episode at 12 months. They found that amygdala volumes were smaller in preterm infants than in full-term infants. They also observed that amygdala volumes were larger in infants showing an escape response of fear compared to the infants showing no escape response. In other words, 12-month-old preterm infants display a reduced fear reactivity and it seems to be related to smaller amygdala volumes. Our results suggest that music listening in the NICU could have some positive effects on fear processing and regulation and it could also have a positive impact on amygdala volumes or related connected brain regions. It would be interesting to address this question in further studies.

Secondly, during the anger-eliciting episode of the Lab-TAB, the 24-month-old full-term group expressed a higher level of anger reactivity than the preterm-control and the preterm-music groups during the second trial. A previous study using the same task found contradictory results with no significant difference at 24 months observed between full-term and preterm children (Lejeune et al., 2015). Nevertheless, it should be noted that the present study used corrected age, while the previous one used the chronological age. This difference could explain the discrepancy between the results. More importantly, our results indicated that the difference of anger reactivity was less pronounced between the preterm-music and the full-term groups ($\eta_{p}^{2}$ = 0.39), than between the preterm-control and the full-term groups ($\eta_{p}^{2}$ = 0.45). It seems that the music intervention would also have a positive impact on the processing of anger with an anger reactivity in the preterm-music group closer to that of the full-term group.

It is also interesting to note that the significant group differences for fear and anger regulation were observed only during the second trial of the episodes. It is possible that preterm children had greater difficulties in maintaining an optimal level of emotional processing and regulation when the emotion-eliciting episodes were repeated. Previous findings indicated differences in emotion regulation strategies between preterm and full-term children (Clark et al., 2008; Evrard et al., 2011). Preterm children could have altered emotion regulation strategies that did not allow for optimal emotional regulation over time. It would explain why significant differences appeared between preterm and full-term children only in the second trial, and consequently why the potential positive impact of music intervention could only be observed in the second trial.

In addition, the three groups did not significantly differ for the cognitive, language, and motor scales of the BSID-III. This result contrasts with previous researches showing that preterm infants achieved lower mean scores on all of the Bayley-III scales than full-term ones at 12 and 24 months of age (Yu et al., 2013; Gasparini et al., 2017). Finally, no significant group difference was found for joy, sustained attention and inhibition abilities. Previous studies found different results with higher levels of joy reactivity in 12-month-old preterm children, different attention scores in 12- and 24-month-old preterm children compared to full-term children, and inhibition difficulties at 24 months (Langerock et al., 2013; Lejeune et al., 2015). The small sample size of the present study could explain these differences of result. Future studies with a larger sample are necessary to verify these preliminary findings.

Preterm children and adolescents are at greater risk for emotional problems (Johnson and Marlow, 2011). Indeed, numerous studies reported a higher prevalence of internalizing problems in this population with an increased risk for anxiety symptoms, depressive symptoms, and withdrawn behavior (Guedeney et al., 2012; Somhovd et al., 2012; Montagna and Nosarti, 2016). Dimitrova et al. (2018) have recently shown that early emotional problems in 18-month-old preterm children predicted later internalizing problems at 11 years of age, but this link was moderated by the severity of perinatal stress. Preterm children who experienced high perinatal stress were at increased risk for emotional difficulties during preadolescence. Emotional problems also seem to persist into adulthood in the preterm population (Montagna and Nosarti, 2016). Our results regarding fear and anger processing and regulation suggest that music listening in the NICU may moderate the effects of preterm birth on later emotion mechanisms, especially in the more vulnerable preterm infants.

In addition, other factors may affect cognitive and emotional development, such as mother-child interaction, maternal anxiety and maternal sensitivity (Forcada-Guex et al., 2006; Zelkowitz et al., 2011; Bouvette-Turcot et al., 2017). For example, among the possible mother-infant dyadic patterns of interaction, the controlling pattern (with a controlling mother and a compliant child) was more often observed among preterm than full-term dyads at 6 months of age and was related to poorer developmental outcome at 18 months of age (Forcada-Guex et al., 2006). Interestingly, a recent study suggests that high maternal sensitivity during mother-infant interaction when the infant was 18 months old is a long-term resilience factor that prevents the development of internalizing disorders in 11-year-old preterm children (Faure et al., 2017). These studies highlight the importance of considering mother-child interaction, parental anxiety and maternal sensitivity as factors to control in future studies.

Introducing music in the NICU had positive effects on brain development in preterm infants. Indeed, Lordier et al. (2018) showed that listening to a familiar music every day during the NICU stay enhanced preterm infants’ connectivity between the right primary auditory cortex and the left caudate nucleus and between the primary auditory cortices and the left putamen and the superior temporal gyrus at term-equivalent age. This result might reflect that they recognized the music but also perceived it as more arousing and pleasant. Koelsch (2014) indicated that music elicited changes in the cerebral regions underlying emotion (limbic and paralimbic areas) in adults, similarly to full-term newborns (Perani et al., 2010). Moreover, music listening had also positive effects on stress and anxiety reduction, suggesting that it improved emotion regulation abilities (Van Goethem and Sloboda, 2011; Linnemann et al., 2015a; Panteleeva et al., 2018). Our preliminary findings are consistent with the literature supporting that music listening has positive effects in emotion processing and regulation. For the first time, the present study suggests some positive long-term effects of music listening in the NICU on neurodevelopmental outcomes in preterm children, and more specifically on emotion mechanisms.

There is a lack of precise guidelines for the choice of music for newborns: live or recorded, instrumental music or parents singing? Live music has been shown to have a larger effect on heart rate and sleep than recorded music (Garunkstiene et al., 2014). Furthermore, one main advantage of live music is that the musician can adapt his music to the baby’s reactions. However, the use of live music needs a musician to be present for each baby and at the right time, leading to some difficulties to conduct live intervention during a long duration in NICUs and standardization of the music intervention becomes impossible. A second major concern in these developmental care interventions is the involvement of parents. Indeed, developmental care programs have shed light on the importance for the parents to be partners in their infant’s care (Craig et al., 2015; Bieleninik et al., 2016). Recently, two reviews described physiologic and behavioral stabilization effect of maternal voice intervention in NICU care (Filippa et al., 2017; Provenzi et al., 2018). It is however important to mention that the present music intervention does not aim to replace the maternal presence/voice, but rather to complete its beneficial effects. Indeed, it is not possible for all mothers to be present every day with their baby in the NICU (for example, other children to take care). Future studies should compare the effect of mother speaking/singing versus music intervention on neurodevelopmental outcomes in preterm infants.

Limitations to the generalizability of our findings should be addressed. First, it included a relatively small sample size. Second, the attrition rate was quite important. Moreover, not all children could be assessed at both ages: some could only be seen at 12 months and others only at 24 months. This reveals the great difficulty of conducting longitudinal follow-up studies. Finally, Bonferroni corrections could also have been conducted given the multiple comparisons. However, since this exploratory study presented preliminary results with a relatively modest sample size, we did not perform such a threshold correction. From our data, we performed a calculation of the sample size (with power goal = 0.8) needed for a randomized controlled trial to answer the original research question, i.e., does music exposure in the NICU have an effect on emotional regulation (Unpredictable toy at 12 months)? These analyses indicate that it will be necessary to include at least 52 children (intervention and control groups). In their recent review of literature, Anderson and Patel (2018) underlined “the pressing need to examine the long-term neurodevelopmental outcomes of children who undergo music interventions in the NICU.” The present study provides preliminary, but promising, scientific findings on the beneficial long-term effects of the music intervention in preterm children. However, future studies are needed with larger number of participants, in order to confirm and complement our results.

## Conclusion

In conclusion, for the first time, the current study suggests some positive long-term effects of music listening in the NICU on emotion processing and regulation at 12 and 24 months of age in preterm children. Our findings bring new insights for supporting music intervention in the NICU. It would be interesting to investigate the later emotion processing of these infants in order to know if this positive effect would persist during childhood.

## Data Availability

All data of this study are included in the manuscript.

## Author Contributions

FL and LL conceptualized and designed the study, collected the data, performed and interpreted data analyses, and drafted the initial manuscript. MP and LS collected the data. DG conceptualized and designed the study, critically reviewed, and revised the manuscript. PH obtained funding for the study, conceptualized and designed the study, critically reviewed, and revised the manuscript. MF interpreted data analyses and drafted the initial manuscript. CBT conceptualized and designed the study, interpreted data analyses, and drafted the initial manuscript. All authors approved the final manuscript as submitted.

## Conflict of Interest Statement

The authors declare that the research was conducted in the absence of any commercial or financial relationships that could be construed as a potential conflict of interest.

## Footnotes

1 http://vollenweider.com/en
